# Stool frequency and form and gastrointestinal symptoms differ by day of the menstrual cycle in healthy adult women taking oral contraceptives: a prospective observational study

**DOI:** 10.1186/s12905-020-01000-x

**Published:** 2020-06-29

**Authors:** Taylor C. Judkins, Jennifer C. Dennis-Wall, Shireen Madani Sims, James Colee, Bobbi Langkamp-Henken

**Affiliations:** 1 Food Science and Human Nutrition Department, University of Florida, 572 Newell Dr, Gainsville, FL 32611 USA; 2 Sensical Science, LLC, 3104 Sneed St #316, Dallas, TX 75204 USA; 3 University of Florida College of Medicine, 3056 SW 93rd Street, Gainesville, FL 32608 USA; 4 IFAS Statistical Consulting Unit, University of Florida, PO Box 110500, Gainesville, FL 32611-0500 USA

**Keywords:** Women’s health, Gastrointestinal, Menstruation

## Abstract

**Background:**

Little is known about how the menstrual cycle affects gastrointestinal function and self-reported stress in young, healthy women taking oral contraceptives (OC). This study prospectively characterized gastrointestinal function and symptoms on each day throughout the menstrual cycle.

**Methods:**

Healthy women aged 18–35 years (*n* = 78) who took OC participated in the 5-week observational study. Stool frequency, self-reported stress, stool form measured by the Bristol Stool Form Scale (BSFS), and gastrointestinal symptoms measured by a modified version of the Gastrointestinal Symptom Rating Scale (GSRS) were assessed daily. GSRS scores were reported (1 = no discomfort at all, 7 = very severe discomfort) and were averaged for individual syndrome scores or summed for the total score. The validated, weekly version of the GSRS was completed at two time points to reflect menstruation and 1 week prior to menstruation (*n* = 72). Outcomes were analyzed in linear mixed models with the Dunnett’s post hoc test against day 1 of menstrual bleeding or with nonparametric tests.

**Results:**

Daily stress (*P* = 0.0018), BSFS score (*P* = 0.0493), stool frequency (*P* = 0.0241), abdominal pain (*P* < 0.0001), diarrhea (*P* = 0.0022), constipation (*P* = 0.0446), reflux (*P* = 0.0193), and indigestion (*P* < 0.0001) all varied significantly by the day of the menstrual cycle. Dunnett’s post hoc tests showed that scores (mean ± SEM) on the first day of bleeding (day 1) for daily abdominal pain (2.6 ± 0.2), diarrhea (1.7 ± 0.1), and indigestion (2.1 ± 0.2) symptoms were higher than scores on all other days of the menstrual cycle (*P* < 0.05) with scores not on day 1 falling under 1.5, or between no discomfort at all and slight discomfort. Reflux, stool frequency, BSFS, self-reported stress, and constipation were higher on day 1 (*P* < 0.05) than on 12, 8, 6, 4, and 2 other days of the menstrual cycle, respectively. The median (IQR) GSRS score was higher during the week of menstruation than the week prior to menstruation for diarrhea [1.50 (1.00–2.33) vs 1.33 (1.00–2.00), *P* = 0.002] and abdominal pain [2.00 (1.33–2.67) vs 1.67 (1.33–2.33), *P* = 0.011] syndrome scores.

**Conclusion:**

Bowel habits appear to vary across the menstrual cycle and suggest more gastrointestinal discomfort on day 1 of menstrual bleeding in healthy women taking OC. Future interventional studies could identify ways to improve gastrointestinal symptoms in healthy women during menstruation.

## Background

Researchers have begun to spend a considerable amount of time characterizing bowel habits of different populations [1–7] with the ultimate goal of treating or preventing symptoms associated with abnormal or undesirable bowel function. In the process, gender differences in gastrointestinal (GI) function have emerged [8–10], particularly in younger women [7]. The reasons for these differences are not clear but have been hypothesized in some studies to be related to the menstrual cycle [9]. Hormone fluctuations from the menstrual cycle influence a multitude of processes, including sensory perception [11–13], sexual desire [14], cognitive function [15], and emotional processing [15]; however, few studies have examined the relationship these hormones have with the GI system. Understanding any mechanisms linking menstruation to bowel discomfort will aid in the development of targeted treatments and potentially provide a substantial impact on the quality of life and/or well-being of women who experience discomfort during and around menstruation. Additionally, measurement tools used in research to capture related data could be refined.

During the days leading up to the onset of natural cycle menstruation, estrogen and progesterone are produced by the ovaries in preparation for a possible pregnancy, and then are sustained throughout if a pregnancy occurs. These hormones are known to slow GI motility [16–18], often resulting in constipation that is commonly experienced during pregnancy [19]. At menstruation, there is a sudden drop in these hormones and a simultaneous spike in prostaglandins, which contract local smooth muscle tissue during menstruation [20]. Because of the close proximity of the uterus to the intestinal tract, it has been hypothesized that these endogenous compounds result in constipation during the days leading up to menstruation (due to progesterone) and diarrhea or loose stools during the first few days of menstruation (due to prostaglandins) [21]. This process is altered by the use of oral contraceptives (OC). Combination OC contain a synthetic estrogen and a progestin that work synergistically to suppress ovulation, thin the endometrium, and reduce natural cycle hormonal fluctuations of ovarian estrogen and progesterone. Typically, a pack of OC contains 21 days of hormone pills followed by 7 days of inert placebo pills that result in a withdrawal bleed. The thin endometrium results in reduced production of prostaglandins; however, prostaglandin fluctuations still occur to some degree, and also play a role in menstruation in women taking OC [22].

Measures of bowel habits and discomfort, such as stool frequency, intestinal transit time, abdominal pain, and constipation, with respect to the menstrual cycle phases have been characterized in women with irritable bowel syndrome [9, 23] and dysmenorrhea [24], but such studies are lacking in healthy women. One study characterized emotional symptoms and aspects of GI discomfort in healthy women around the time of menstruation [25], but these discomforts were measured with retrospective recall tools and did not use available validated measures such as the Bristol Stool Form Scale (BSFS) [26, 27] or the Gastrointestinal Symptom Rating Scale (GSRS) [28] as that was not the objective of the exploratory study. Such measurements are required in prospective studies to identify events occurring as a function of the menstrual cycle. As previously stated, it is also well established that the menstrual cycle affects cognitive processing and emotional well-being, as increased rates of anxiety and depression, as well as other psychiatric symptoms, are described in the literature [29–31]. Additionally, few if any studies have examined the relationship between self-reported stress and GI symptoms during the menstrual cycle in healthy women taking OC.

The primary objective of this study was to determine the effect of the menstrual cycle in women taking OC on GI habits and symptoms using validated measures of GI function. It was hypothesized that both the average BSFS scores as a validated proxy for intestinal transit time [26, 27] and average stool frequency would differ across the menstrual cycle. Self-reported daily stress and GI symptoms from the GSRS were also expected to differ during the first day of bleeding and were analyzed as secondary outcomes.

## Methods

### Study participants

**Fig. 1:**
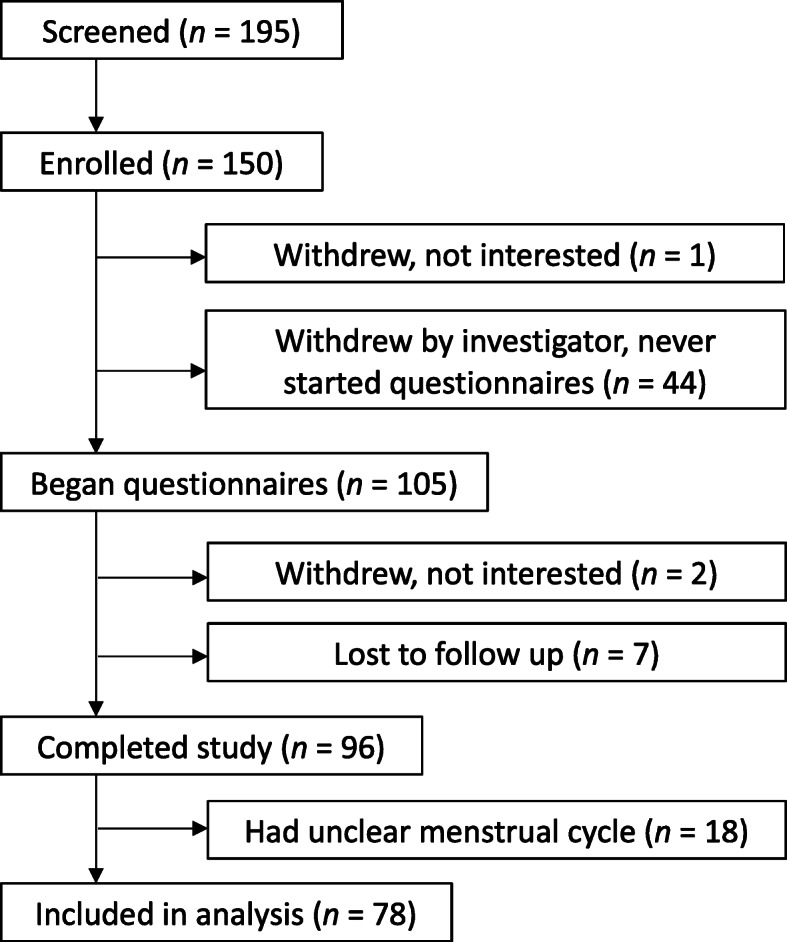
Study flow diagram

Healthy females aged 18 to 35 years were recruited for this study (Fig. 1). The age range was selected in order to exclude women who may have premature menopause [32]. Potential participants were recruited by distributing printed flyers in a community in Florida, sending emails via university listservs, and posting on social media. Participation was open to anyone as long as they had internet access and met the inclusion criteria. After providing informed consent online using methods approved by the University of Florida’s Institutional Review Board (IRB201701246), participants self-screened based on the following criteria: 1) must be regularly menstruating (i.e., ≥12 times/year), 2) must be willing and able to maintain their normal diet and exercise habits for the duration of the study, 3) must not be lactating or knowingly pregnant, 4) must not be receiving treatment for a physician-diagnosed gastrointestinal disease or condition, and 5) must be taking OC. Only women on OC were included to reduce the chance of enrolling women with irregular menstrual cycles and to better align the daily data based on a more predictable cycle length. Including women who are on OC (28-day cycle) also allowed for the administration of validated weekly questionnaires at designated time points.

### Data collection and questionnaires

This was a prospective, 5-week observational study. After consenting, participants self-reported height, weight, and typical fibre intake using a fruit, vegetable, and fibre screener (NutritionQuest) [33]. Participants were asked to contact a member of the research team on the first day of their menstrual cycle so they could begin answering daily questionnaires. Daily questionnaires were administered to assess stool frequency (number of bowel movements per day), stool form (as measured by the BSFS), self-reported daily stress levels (0 = no stress to 10 = extremely stressed), and whether or not the participant was currently menstruating. The GSRS, which asks about GI symptoms over the past 7 days, was administered on day 7 of the menstrual cycle to capture the first week following the start of menstruation (days 1 to 7) and again on day 26 to capture the days leading up to menstruation (days 19 to 25 assuming a 28-day cycle). Although the GSRS is validated to measure bowel discomfort in diseased populations, this tool has identified significant differences in outcomes between both intervention groups [6] and genders [34] in intervention studies. For the purposes of measuring these symptoms in women throughout the menstrual cycle, the 7-day recall window in the validated version of the GSRS may be too long to capture important changes that occur on a single day, such as the first day of menstruation or the day before menstruation. Therefore, a modified version of the GSRS (see Additional file 1) was also asked daily as an exploratory outcome. Questionnaires were sent in the mornings and asked about the previous day so that answers would reflect all waking hours. A study coordinator monitored questionnaire completion daily and sent reminder emails or made phone calls when necessary.

### Statistical analysis

#### Sample size and ethical considerations

A convenience sample of 150 women was obtained between September 6 and November 2 of 2017. This study was reviewed and approved by the University of Florida Institutional Review Board 02. Informed consent was obtained from all participants prior to any study activities.

#### Day determination and renumbering

All participants began daily questionnaires on their first day of menstruation and continued for 5 weeks unless they withdrew or were lost to follow up. The study spanned two menstrual events for the purpose of capturing pre-menstrual days leading up to a menstrual event, thus the first day of their second menstrual event (i.e., the first of at least 2 days of answering “yes” to the question “did you menstruate yesterday?”) was designated as day 1. Days of participant data were numbered forward from day 1 to day 10. This allowed for questionnaire data to be aligned by day of the menstrual cycle to characterize any cyclic patterns in GI symptoms and habits. To align data from women with a different number of days in their menstrual cycle, days were numbered in reverse order from day − 1 (i.e., day before first day of bleeding) to day − 15 except when the second menstrual event could not be identified during the period of observation (i.e. menstrual cycle was ≥35 days) or when the cycle length was < 24 days [35]. In these cases, data could not be aligned and were not included in the daily outcome analyses. Only 25 days were analyzed to capture 10 days after the time of bleeding. After day 10 the number of observations began to decline due to variation in cycle lengths between participants.

Data for GSRS outcomes were included for any participant who had a cycle length ≥ 26 days from the first menstrual event so that day 26 (i.e., the second time the GSRS was administered) represented pre-menstrual days.

#### Study outcomes

Stool frequency, BSFS score, self-reported stress, and all modified GSRS daily outcomes were each analyzed as the response variables in linear mixed models with a fixed effect of day and a heterogeneous autoregressive covariance structure for repeated measures on the same individual to assess effects by day of menstrual cycle. To meet assumptions of normality, all outcomes were log-transformed for analysis except stool frequency and self-reported stress, which underwent square root transformations due to having values equal to zero. Day 1 visually appeared to differ from all other days and was suspected to be the reason for day effects, so post hoc Dunnett’s tests were completed for each day against day 1 for all daily outcomes.

GSRS syndrome scores during menstruation and the days leading up to menstruation (days − 9 to − 3) for all but indigestion syndrome were not normally distributed and were thus compared by using Wilcoxon Signed Rank tests. Indigestion syndrome scores were compared using a paired t-test.

Sigma Plot v12.5 (Systat Software Inc., San Jose, CA, USA) and SAS v9.4 (SAS Institute Inc., Cary, NC, USA) were used for all analyses. Significance of statistical tests was determined using a type I error rate cut-off of 0.05. Unless stated otherwise, data are reported as the means ± SD.

## Results

Participants began questionnaires within a two-month timeframe. Of the participants who completed 5 weeks of questionnaires, 78/96 (81%) were included in the analyses of daily outcomes, and 72/96 (75%) were included in the GSRS outcome analysis. Participants on average had a body mass index within the healthy range [23.4 ± 3.1 kg/m^2^], a typical fibre intake of 14.8 ± 3.8 g/day, and a menstrual cycle length of 28.2 ± 1.8 days.

**Fig. 2:**
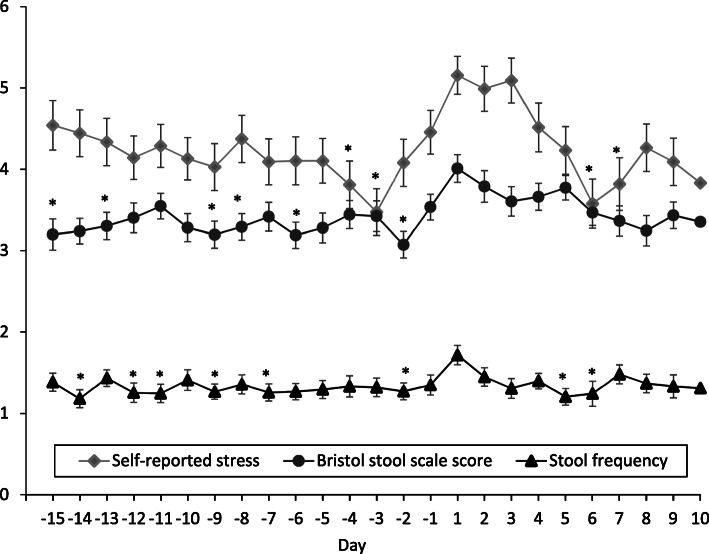
Stool frequency, stool form, and self-reported daily stress differed by day of menstrual cycle (*P* < 0.05). Scales and definitions for outcomes: stool frequency, number of bowel movements per day; stool form, Bristol stool form scale score (1 = slower intestinal transit, 7 = faster intestinal transit); self-reported daily stress (0 = no stress, 10 = extremely stressed). Data represent the means±SEM. Twenty-five days were analyzed to capture the theoretical day of ovulation through day 10 post-menstruation. After day 10 the number of observations began to decline. Day 1 represents the first day of menstruation. The significant effect of day was determined by using generalized linear mixed models. Post hoc Dunnett’s tests for multiple comparisons identified days of interest (*P < 0.05 vs Day 1)

**Fig. 3:**
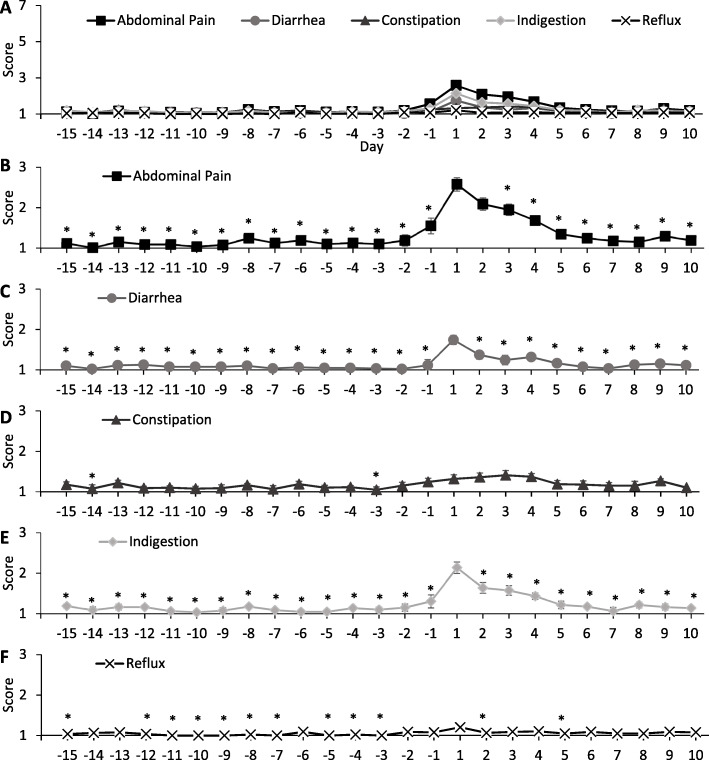
Modified daily Gastrointestinal Symptom Rating Scale scores differed by day of menstrual cycle (*P* < 0.05). 1 = no discomfort at all, 7 = very severe discomfort for abdominal pain (abdominal pain, hunger pains, and nausea), diarrhea (diarrhea, loose stools, and urgent need for defecation), constipation (constipation, hard stools, and feeling of incomplete evacuation), indigestion (rumbling, bloating, burping, and gas), and reflux (heartburn and acid regurgitation) syndromes. Panel A depicts all scores together on the scale used in the questionnaire; panels B-F depict individual syndrome scores with results from post hoc tests. Data represent the means±SEM. Day 1 represents the first day of menstruation. The significant effect of day was determined by using generalized linear mixed models. Post hoc Dunnett’s tests for multiple comparisons identified days of interest (*P < 0.05 vs Day 1)

Stool frequency (*P* = 0.0241), BSFS (*P* = 0.0493), and self-reported stress (*P* = 0.0018) all differed by day and peaked on day 1 (Fig. 2). When daily GSRS items were analyzed by day, abdominal pain (*P* < 0.0001), diarrhea (*P* = 0.0022), constipation (*P* = 0.0446), indigestion (*P* < 0.0001), and reflux (*P* = 0.0193) symptoms all differed by day and peaked on day 1 except for constipation, which peaked on day 3 (Fig. 3). For GSRS data that were measured at the two time points, the median (IQR) GSRS score was higher during the week of menstruation than during the pre-menstrual week for total [27 (22–36) vs 26 (20–33), *P* = 0.002], diarrhea [1.50 (1.00–2.33) vs 1.33 (1.00–2.00), *P* = 0.002], and abdominal pain [2.00 (1.33–2.67) vs 1.67 (1.33–2.33), *P* = 0.011] syndrome scores. Reflux, constipation, and indigestion syndrome scores were not different between time points.

## Discussion

The relationship between GI discomfort and menstruation has been established in some populations but is underexplored in healthy women (i.e. those who have not been diagnosed with GI diseases or conditions) taking OC. A previous systematic review reported that one-third of women report at least one GI symptom during menstruation, however, these data did not separate women who do and do not consume OC and included data that asked women to retrospectively report their typical symptoms over their cycle [36]. This prospective study revealed a relationship between many GI outcomes and the first day of the menstrual cycle. To our knowledge, this is also the first study to prospectively use the well-accepted measures of stool frequency, as an indicator of laxation [37], and BSFS, as a validated indicator of intestinal transit time [26, 27], to assess bowel function in this population.

In the current study, stool frequency, BSFS score, self-reported stress, and all symptoms measured with a modified daily version of the GSRS except for constipation were higher on day 1 than on many other days of the menstrual cycle. Although previous studies did not use validated tools to measure similar outcomes, these findings support previous reports that women who take oral contraceptives experience looser stools [24] and more severe GI symptoms during menses [23]. Overall, the modified GSRS scores were of physiological significance in that participants moved from no discomfort to slight/mild discomfort from GI symptoms. The day-to-day change in these symptoms, as well as stool frequency and BSFS score, in relation to the menstrual cycle may reflect the cyclic changes in reproductive hormones and/or prostaglandins, whether endogenous or synthetic. It was anticipated that there would be a slowing of intestinal transit before menstruation due to progesterone, as OCs are progesterone dominant, and a speeding of transit and induction of GI muscle contraction during menstruation due to prostaglandins released from the endometrium. Accordingly, BSFS scores were lowest on day − 2, indicating that intestinal transit time was slowest in the days prior to menstruation. This is in accordance with a previous study that demonstrated the colonic transit time is significantly longer during the luteal phase than during the follicular phase; however, transit time was averaged over phases rather than days of the menstrual cycle [17]. Additionally, it is unknown if this population was on OC or not [17]. Constipation scores measured by the modified GSRS were not the highest on this day as would be expected, indicating that these objective and subjective measures of bowel function are not perfectly correlated. Finally, self-reported stress was the highest on day 1 of menstruation, indicating that quality of life, while not measured in this study, may also be negatively affected during menstruation. The increase in stress could be due to the inconveniences and/or mood changes [25, 38] experienced by women while menstruating and/or to the GI disturbances that were demonstrated in this study, which is likely as stress and GI symptoms are closely related [34]. As with any observational study, only associations have been presented here, and cause and effect thus cannot be established.

The generalizability of these results depends on how well the study sample represents the general population. In this study, participants were between 18 and 35 years of age, had a typical and healthy body mass index of about 23 kg/m^2^, had a low but typical [39] fibre intake of about 15 g/day, and had a typical menstrual cycle length of about 28 days. Since these variables may influence GI function and were also typical of the general population, we argue that the results can likely be generalized to young, healthy women who take OC. Approximately 62% of women in the United States use some form of contraception and 28% of those women use OC, which is a substantial portion of the population [40]. Exclusion criteria were limited in order to increase the external validity of the study.

### Strengths and limitations

To our knowledge, this is the first study to prospectively collect stool frequency, stool form, and GI symptom data on a daily basis throughout the menstrual cycle in healthy women taking OC. Previous studies asked participants to recall data over the past month [25], which is subject to bias and inaccuracy and is appropriate only in certain situations such as exploratory studies or when prospective measurement is not feasible. We present data by day rather than averaged over a time period, thus providing the detail that has proven to be important as day 1 was different from many other days of the menstrual cycle. This study was specifically designed to capture outcomes at the same time points in the cycle (i.e. surveys were administered to all participants on the same day of their cycle) rather than on the same calendar dates.

Because of the observational nature of the study, confounding is a possible limitation with such factors as stress, fibre intake, medication use, physical activity, or other lifestyle factors. Stress and fibre were expected to potentially influence the outcomes of this study and were thus measured in an attempt to control for them; however, fibre was only measured once at the beginning of the study and could not be analyzed with the outcomes on a daily or weekly basis. Medication use, such as nonsteroidal anti-inflammatory drugs, may impact gastrointestinal symptoms, including indigestion [41]. This information was not recorded. Participants also self-enrolled and were entrusted to read the inclusion criteria carefully before consenting to participate. OC vary widely in application and mechanism, and the dose, formulation, and regimen of OC were not assessed in this study. Days in which the active pills were consumed were also not recorded. It is unlikely that use of OC would increase GI symptoms as these side effects are rare and are typically found in women over 35 years old [42] and this age group was excluded from the study. Use of OC may actually alleviate the severity of GI symptoms, as they are known to control abdominal cramping due to decreased prostaglandin production by the endometrium [43, 44]. In this present study, significant abdominal pain was still observed and could perhaps be more severe in women not taking OC, although this has yet to be measured. Abdominal pain due to menstrual cramps and abdominal pain due to gastrointestinal symptoms during the time of bleeding may also be difficult to differentiate.

## Conclusions

Bowel habits appear to differ between phases of the menstrual cycle and suggest more discomfort on day 1 of menstruation in healthy women taking OC. Future studies should assess whether these GI changes occur in women not taking OC and whether they negatively affect quality of life using validated digestion-associated quality of life tools. The positive results from this study indicate the need to further investigate mechanisms behind these findings, and, accordingly, dietary,lifestyle, and/or extended cycle pill interventions may subsequently be identified to reduce burdens imposed by menstruation-associated GI changes.

## Supplementary information

**Additional file 1.** Modified daily Gastrointestinal Symptom Rating Scale (GSRS) as it appeared in the daily questionnaire. A portion of the daily survey in the form in which it was administered to participants.

## Data Availability

The datasets used and/or analyzed during the current study are available from the corresponding author upon reasonable request and approval of the University of Florida.

## Abbreviations

BMI: Body mass index
BSFS: Bristol stool form scale
GI: Gastrointestinal
GSRS: Gastrointestinal Symptom Rating Scale
OC: Oral contraceptives

## References

[1] Walter SA, Kjellstrom L, Nyhlin H, Talley NJ, Agreus L (2010). Assessment of normal bowel habits in the general adult population: the Popcol study. Scand J Gastroenterol.

[2] Longstreth GF, Thompson WG, Chey WD, Houghton LA, Mearin F, Spiller RC (2006). Functional bowel disorders. Gastroenterology..

[3] Tillisch K, Labus JS, Naliboff BD, Bolus R, Shetzline M, Mayer EA (2005). Characterization of the alternating bowel habit subtype in patients with irritable bowel syndrome. Am J Gastroenterol.

[4] Drossman DA, Morris CB, Hu Y, Toner BB, Diamant N, Leserman J (2005). A prospective assessment of bowel habit in irritable bowel syndrome in women: defining an alternator. Gastroenterology..

[5] Drossman DA, Sandler RS, McKee DC, Lovitz AJ (1982). Bowel patterns among subjects not seeking health care: use of a questionnaire to identify a population with bowel dysfunction. Gastroenterology..

[6] Dennis-Wall JC, Culpepper T, Nieves C, Rowe CC, Burns AM, Rusch CT (2017). Probiotics (Lactobacillus gasseri KS-13, Bifidobacterium bifidum G9-1, and Bifidobacterium longum MM-2) improve rhinoconjunctivitis-specific quality of life in individuals with seasonal allergies: a double-blind, placebo-controlled, randomized trial. Am J Clin Nutr.

[7] Heaton KW, Radvan J, Cripps H, Mountford RA, Braddon FE, Hughes AO (1992). Defecation frequency and timing, and stool form in the general population: a prospective study. Gut..

[8] Higgins PD, Johanson JF (2004). Epidemiology of constipation in North America: a systematic review. Am J Gastroenterol.

[9] Adeyemo MA, Spiegel BM, Chang L (2010). Meta-analysis: do irritable bowel syndrome symptoms vary between men and women?. Aliment Pharmacol Ther.

[10] Degen LP, Phillips SF (1996). Variability of gastrointestinal transit in healthy women and men. Gut..

[11] Caruso S, Mauro D, Maiolino L, Grillo C, Rapisarda AMC, Cianci S (2018). Effects of combined oral contraception containing drospirenone on premenstrual exacerbation of Meniere's disease: preliminary study. Eur J Obstet Gynecol Reprod Biol.

[12] Serra A, Maiolino L, Agnello C, Messina A, Caruso S (2003). Auditory brain stem response throughout the menstrual cycle. Ann Otol Rhinol Laryngol.

[13] Al-Mana D, Ceranic B, Djahanbakhch O, Luxon LM (2008). Hormones and the auditory system: a review of physiology and pathophysiology. Neuroscience..

[14] Caruso S, Agnello C, Malandrino C, Lo Presti L, Cicero C (2014). Cianci. Do hormones influence women's sex? Sexual activity over the menstrual cycle. J Sexual Med.

[15] Sundstrom Poromaa I, Gingnell M (2014). Menstrual cycle influence on cognitive function and emotion processing-from a reproductive perspective. Front Neurosci.

[16] Li Y, Yu Y, Li S, Zhang M, Zhang Z, Zhang X (2018). Isobaric tags for relative and absolute quantification-based proteomic analysis that reveals the roles of progesterone receptor, inflammation, and fibrosis for slow-transit constipation. J Gastroenterol Hepatol.

[17] Jung HK, Kim DY, Moon IH (2003). Effects of gender and menstrual cycle on colonic transit time in healthy subjects. Korean J Intern Med.

[18] Xiao ZL, Pricolo V, Biancani P, Behar J (2005). Role of progesterone signaling in the regulation of G-protein levels in female chronic constipation. Gastroenterology..

[19] Baron TH, Ramirez B, Richter JE (1993). Gastrointestinal motility disorders during pregnancy. Ann Intern Med.

[20] Evans J, Salamonsen LA (2012). Inflammation, leukocytes and menstruation. Rev Endocr Metab Disord.

[21] Heitkemper MM, Chang L (2009). Do fluctuations in ovarian hormones affect gastrointestinal symptoms in women with irritable bowel syndrome?. Gend Med.

[22] Garg SK (1983). Serum prostaglandin F levels during menstrual cycle in women using oral contraceptives. Int J Clin Pharmacol Ther Toxicol.

[23] Heitkemper MM, Cain KC, Jarrett ME, Burr RL, Hertig V, Bond EF (2003). Symptoms across the menstrual cycle in women with irritable bowel syndrome. Am J Gastroenterol.

[24] Heitkemper MM, Shaver JF, Mitchell ES (1988). Gastrointestinal symptoms and bowel patterns across the menstrual cycle in dysmenorrhea. Nurs Res.

[25] Bernstein MT, Graff LA, Avery L, Palatnick C, Parnerowski K, Targownik LE (2014). Gastrointestinal symptoms before and during menses in healthy women. BMC Womens Health.

[26] Blake MR, Raker JM, Whelan K (2016). Validity and reliability of the Bristol stool form scale in healthy adults and patients with diarrhoea-predominant irritable bowel syndrome. Aliment Pharmacol Ther.

[27] Lewis SJ, Heaton KW (1997). Stool form scale as a useful guide to intestinal transit time. Scand J Gastroenterol.

[28] Revicki DA, Wood M, Wiklund I, Crawley J (1998). Reliability and validity of the gastrointestinal symptom rating scale in patients with gastroesophageal reflux disease. Qual Life Res.

[29] Strine TW, Chapman DP, Ahluwalia IB (2005). Menstrual-related problems and psychological distress among women in the United States. J Women’s Health.

[30] Bowen R, Bowen A, Baetz M, Wagner J, Pierson R (2011). Mood instability in women with premenstrual syndrome. J Obstet Gynaecol Can.

[31] Lane T, Francis A (2003). Premenstrual symptomatology, locus of control, anxiety and depression in women with normal menstrual cycles. Arch Womens Ment Health.

[32] Okeke TC, Anyaehie UB, Ezenyeaku CC (2013). Premature Menopause. Ann Med Health Sci Res.

[33] NutritionQuest. Block Fruit/Vegetable/Fiber Screener. 2014. [http://nutritionquest.com/wellness/free-assessment-tools-for-individuals/fruit-vegetable-fiber-screener/]. Accessed 1 Oct 2018.

[34] Culpepper T, Christman MC, Nieves C, Specht GJ, Rowe CC, Spaiser SJ (2016). Bifidobacterium bifidum R0071 decreases stress-associated diarrhoea-related symptoms and self-reported stress: a secondary analysis of a randomised trial. Benef Microbes.

[35] Fraser IS, Critchley HO, Broder M, Munro MG (2011). The FIGO recommendations on terminologies and definitions for normal and abnormal uterine bleeding. Semin Reprod Med.

[36] Moore J, Barlow D, Jewell D, Kennedy S (1998). Do gastrointestinal symptoms vary with the menstrual cycle?. Br J Obstet Gynaecol.

[37] Guidance for industry: scientific evaluation of the evidence on the beneficial physiological effects of isolated or synthetic non-digestible carbohydrates submitted as a citizen petition (21 CFR 10.30). U.S. Food and Drug Administration Center for food safety and applied nutrition. 2018. https://www.fda.gov/food/guidanceregulation/guidancedocumentsregulatoryinformation/ucm528532.htm. Accessed 19 Mar 2019.

[38] Farage MA, Osborn TW, MacLean AB (2008). Cognitive, sensory, and emotional changes associated with the menstrual cycle: a review. Arch Gynecol Obstet.

[39] What We Eat in America NHANES 2013–2014. Nutrient intakes per 1000 kcal from food and beverages: mean energy and mean nutrient amounts per 1000 kcal consumed per individual, by gender and age, in the United States, 2013–2014. 2014. [https://www.ars.usda.gov/ARSUserFiles/80400530/pdf/1314/Table_41_DEN_GEN_13.pdf]. Accessed 31 Jan 2019.

[40] Jones J, Mosher W, Daniels K (2012). Current contraceptive use in the United States, 2006-2010, and changes in patterns of use since 1995. Natl Health Stat Report.

[41] Seager JM, Hawkey CJ (2001). Indigestion and non-steroidal anti-inflammatory drugs. BMJ..

[42] IBM Micromedex. Estrogen and progestin oral contraceptives (oral route). 2019. [https://www.mayoclinic.org/drugs-supplements/estrogen-and-progestin-oral-contraceptives-oral-route/side-effects/drg-20069422]. Accessed 19 Mar 2019.

[43] Dmitrovic R, Kunselman AR, Legro RS (2012). Continuous compared with cyclic oral contraceptives for the treatment of primary dysmenorrhea: a randomized controlled trial. Obstet Gynecol.

[44] Proctor ML, Roberts H, Farquhar CM. Combined oral contraceptive pill (OCP) as treatment for primary dysmenorrhoea. Cochrane Database Syst Rev. 2001;(4):Cd002120. 10.1002/14651858.CD002120 11687142

