# Supplementary material for: Stool frequency and form and gastrointestinal symptoms differ by day of the menstrual cycle in healthy adult women taking oral contraceptives: a prospective observational study
Source: BMC Womens Health. 2020 Jun 29;20:136. doi: 10.1186/s12905-020-01000-x (PMC7325082; doi:10.1186/s12905-020-01000-x)
Supplement: Supplementary file 1 — Additional file 1. Modified daily Gastrointestinal Symptom Rating Scale (GSRS) as it appeared in the daily questionnaire. A portion of the daily survey in the form in which it was administered to participants. [file 12905_2020_1000_MOESM1_ESM.docx]

Modified daily Gastrointestinal Symptom Rating Scale (GSRS) as it appeared in the daily questionnaire.

Did you have any GI Symptoms related to menstruation? (Abdominal pain, diarrhea, constipation, indigestion, or reflux)

- Yes
- No

If so, please indicate which symptoms you experienced due to menstruation

|  | No discomfort at all | Slight discomfort | Mild discomfort | Moderate discomfort | Moderately severe discomfort | Severe discomfort | Very severe discomfort |
| --- | --- | --- | --- | --- | --- | --- | --- |
| **Abdominal Pain** (including abdominal pain, hunger pains, and nausea) |  |  |  |  |  |  |  |
| **Diarrhea** (including diarrhea, loose stools, and urgent need for defecation) |  |  |  |  |  |  |  |
| **Constipation** (including constipation, hard stools, and feeling of incomplete evacuation) |  |  |  |  |  |  |  |
| **Indigestion** (including rumbling, bloating, burping, and gas) |  |  |  |  |  |  |  |
| **Reflux** (heartburn and acid regurgitation) |  |  |  |  |  |  |  |
